# Supplementary material for: SSC: The novel self-stack ensemble model for thyroid disease prediction
Source: PLoS One. 2024 Jan 3;19(1):e0295501. doi: 10.1371/journal.pone.0295501 (PMC10763970; doi:10.1371/journal.pone.0295501)
Supplement: Attachment — Submitted filename: SSC paper response.docx [file pone.0295501.s001.docx]

**RESPONSE TO REVIEWERS**

**Manuscript ID:** PONE-D-22-32955

**Article Title:** “The Novel Self-Stack Ensemble Model for Thyroid Disease Prediction”

**Author:** Ji Sheng-jun

Dear Editor,

First, we are extremely grateful to the editor and all the reviewers who conducted a rigorous and constructive review of our article number PONE-D-22-32955. Certainly, all the review points were very well directed, and we managed to improve our manuscript a great deal and had to work a lot to address all the changes suggested by the reviewers. We have incorporated suggestions and guidance provided by the reviewers and made a careful revision of the manuscript. We have tried our level best to improve this manuscript by organizing and improving our figures to present the material more constructively and effectively. This has certainly, raised the quality of this research work and will make it a valuable contribution to the community. We hope that we have managed to fulfill the required changes suggested by the valuable reviewers.

Please see our responses (in blue color) in line with the reviewers’ comments in black color.

Best regards,

Ji Sheng-jun

**Editor Comments:**

- **Concern 1:** It should be cleared why in figure 9, the sum of the numbers of each confusion matrix is different. For example, the sums of the first lines in the subgraphs (a)(b)(c) is 51, while the first lines of (e)(f) and (j) add up to only 35. This should not occur if the same test set was used in each case.

**Response:** We would like to thank the editor for the valuable comments to improve the quality of the manuscript. You rise a good point and help us to mention it in the manuscript for clarity. We have done separate data splitting (Training and Testing sets) for machine learning deep learning models. When splitting the dataset, we used the shuffle parameter which changes the number of instances for each target class but in both cases, the total numbers of test samples are the same which is 230. We did splitting separately for deep learning models because you can see the last layer of deep learning have 5 neurons so we have to feed target data after converting it into five variables so we did splitting again after the conversion of data for deep learning models and shuffle parameters change the count of for each category in the test dataset.

- **Concern 2:** It should be discussed if RF, SVC and LR models were trained with optimal values of parameters.

**Response**: We would like to thank the reviewer for the valuable comments to improve the quality of the manuscript. We select the best hyperparameter setting by tuning the models on specific value ranges. We added the tuning range of the model's hyperparameters value to discuss the selection of optimal values of parameters for models.

- **Concern 3:** Algorithms should be presented with proper indentation.

**Response**: We make changes in the manuscript according to the reviewer's suggestion. We make corrections in algorithms indentation.

**Reviewer #1:**

**Concern 1:** In figure 9, why the sum of the numbers of each confusion matrix is different, for example, the sum of the first line of RF in the subgraph (a) is 51, while the first line of (j) CNN-LSTM adds up to only 35. This problem should not occur in the same test suite.

**Response:** We would like to thank the reviewer for the valuable comments to improve the quality of the manuscript. You rise a good point and help us to mention it in the manuscript for clarity. We have done separate splitting for machine learning deep learning models. When splitting the dataset we used the shuffle parameter which changes the number of instances for target classes but in both cases, the total numbers of test samples are the same which is 230. We did splitting separately for deep learning models because you can see the last layer of deep learning have 5 neurons so we have to feed target data after converting it into five variables so we did splitting again after the conversion of data for deep learning models and shuffle parameters change the count of for each category in the test dataset.

**Concern 2:** When training models such as 2.RF, SVC and LR, are the optimal parameters selected? if not, what are the considerations? Similarly, what hyper-parameters CNN and LSTM choose and what models they choose need to be supplemented and clarified.

**Response:** We would like to thank the reviewer for the valuable comments to improve the quality of the manuscript. We select the best hyperparameter setting by tuning the models on specific value ranges. We added the tuning range of the model's hyperparameters value to discuss the selection of optimal values of parameters for models.

While for LSTM and CNN models architecture and hyperparameters setting we added in Table 6. This hyperparameter setting is according to the literature. We study the researchers who worked on the same kinds of datasets and we used the same kind of state-of-the-art architectures to achieve significant accuracy.

**Reviewer #2:** 

**Concern 1:** Overall this is an interesting paper: well-written and well-motivated to an important problem of applied ML. The author compares their methods with several other standard ML methods. The authors should indent the presentation of the Algorithms properly and add the descriptions of S and C in the caption.

**Response:** We would like to thank the reviewer for the valuable comments to improve the quality of the manuscript. We add the proper indent in the presentation of the Algorithms and also describe the variables in the algorithms.
