# Supplementary material for: SSC: The novel self-stack ensemble model for thyroid disease prediction
Source: PLoS One. 2024 Jan 3;19(1):e0295501. doi: 10.1371/journal.pone.0295501 (PMC10763970; doi:10.1371/journal.pone.0295501)
Supplement: Attachment — Submitted filename: SSC R3.docx [file pone.0295501.s002.docx]

**RESPONSE TO REVIEWERS**

**Manuscript ID:** PONE-D-22-32955

**Article Title:** “The Novel Self-Stack Ensemble Model for Thyroid Disease Prediction”

**Author:** Ji Sheng-jun

Dear Editor,

First, we are extremely grateful to the editor and all the reviewers who conducted a rigorous and constructive review of our article number PONE-D-22-32955. Certainly, all the review points were very well directed, and we managed to improve our manuscript a great deal and had to work a lot to address all the changes suggested by the reviewers. We have incorporated suggestions and guidance provided by the reviewers and made a careful revision of the manuscript. We have tried our level best to improve this manuscript by organizing and improving our figures to present the material more constructively and effectively. This has certainly, raised the quality of this research work and will make it a valuable contribution to the community. We hope that we have managed to fulfil the required changes suggested by the valuable reviewers.

Please see our responses (in blue color) in line with the reviewers’ comments in black color.

Best regards,

Ji Sheng-jun

Reviewer #1:

Concern: The author replied to my question and gave a better explanation. Based on the revised manuscript, I think it meets the relevant requirements for publication.

**Response**: We would like to thank the reviewer for value comments to improve the quality of manuscript and giving positive feedback.

Reviewer #3:

Concern: Specify what acronyms such as RF stand for. Lectors must knowing what it means when acronym appear at the first time.

**Response**: We would like to thank the reviewer for highlighting an important point and we update manuscript according to reviewer concern. We added the acronyms for all abbreviation.

Reviewer #4:

Concern 1: >>> 1. Language problems:

1.1 "Classifier_base = Base Classifiers i.e RF_base1, RF_base2, and RF_base3" (in Algorithm 2)
"i.e" => "i.e."
We would like to thank the reviewer for highlighting an important point and we update manuscript according to reviewer concern.

Concern 2: >>> 2. Presentation problems:

2.1 Table 11, Table 13, Table 14: units are not presented in the header of the table (accuracy in [%] or [1]). SD for accuracy and other measured values is not given.

**Response**: We would like to thank the reviewer for valuable comments. In Table 11, 13, and 14 and all others Tables we preset results in standard format such as accuracy and other evaluation are between 0 and 1. Our results in Tables are not in %. Second the standard deviation (SD) is output of K-fold cross validation and is present in corresponding Table 13.

Concern 3: 2.2 Fig. 2a and Fig. 2b: most of the names of features near horizontal axis are presented improperly. Those names are cut in a way that makes some of those features indistinguishable (e.g. 6x"sured", 2x"oxine", 2x"yroid". This should not happen.

**Response**: We would like to thank the reviewer for highlighting an important point and we update manuscript according to reviewer concern. We update the manuscript by adding the accurate images.

2.3 Fig. 9, Fig. 5, Fig 6, Fig 2: Results should be presented with error bars.

**Response**: We would like to thank the reviewer for valuable comments.

- Fig 2: its feature importance figure so their cant be any error bar.
- Fig 5 and Fig 6: These two figures consist of accuracy and error graphs, in Fig 5 we illustrate the accuracies graph while in Fig 6 there are error graphs.
- Fig 7 & 8: Both figures consist of confusion matrix which values represent the error rates and accuracies both.
- Fig 9: we added error rate in fig 9 and update the manuscript.

2.4 Table 15: There is no point presenting columns with the same value in all rows of the table. Such information can be given in the title of the table or below the table.

**Response**: We would like to thank the reviewer for valuable comments to improve the quality of manuscript. The P-value column value can be different in each row but in our case it constant. We have T-statistics with P-values to evaluate the performance. So according to situate its better to have value in each row but still if reviewer will suggest we will remove this.

2.5 Algorithm 2:

"D = Number of Classifier_base used." - it is not clear
=>
"D = Number of base classifiers."

**Response**: We would like to thank the reviewer for valuable comments to improve the quality of manuscript. We update manuscript according to reviewer suggestion.

2.6 Fig. 5: what is the meaning of "Val_Accuracy", "Val_Precision", "Val_Recall" and 'Val_F1" within legends of included charts? What is the difference e.g. between "Accuracy' and ""Val_Accuracy"? This should be explained within the title of the figure.

**Response**: We would like to thank the reviewer for the valuable comments to improve the quality of the manuscript. The ‘Accuracy’ represent the accuracy on the training set and ‘Val_Accuracy’ represent the accuracy on the validation set, it is also similar to other evaluation matrixes. We added the description of it in the updated manuscript.

The meanings of 'Accuracy' and 'Val\_Accuracy' in Figures 5 and 6 refer to the accuracy values obtained on the training and validation sets, respectively. The same principle applies to other evaluation measures as well.

2.7 Fig. 6 - analogous problem as the one indicated in point 2.6. What is e.g. Loss and how it is different from Val_Loss?

**Response**: Its very similar to as we mention in response of 2.6, The ‘Loss represent the accuracy on the training set and ‘Val_Loss’ represent the accuracy on the validation set, it is also similar to other evaluation matrixes.

Concern: 3 >>> 3. Other problems:

3.1 Why the time in Table 13 is given with varying precision for different models (please compare results for SVC v.s. GBM v.s. LR)? Were different methods for time measurement used in those cases? It should be specified within the text how the time was measured and what was the precision of this time measurement setup. SD or confdence intervals should be given with measured value.

**Response**: We would like to thank the reviewer for the valuable comments to improve the quality of the manuscript. These all used models are different in their approaches so their computation time can vary on different run. We added the specification of system used for experiments.

All experiments were performed using a Corei7, a 12th-generation Dell machine with a Windows operating system. We used Python language to implement the proposed approach in Jupyter Notebook. Several libraries such as sci-kit learn, TensorFlow, and Keras were used for experimental purposes.

SD for each model is present in Table 13 which show the confidence interval of model.

3.2 Algorithm 2: It is unclear what it the meaning of "mode{}". Maybe Authors meant "model" ?
In both cases it should be clarified how the prediction is generated in the last step of Algorithm 2.

**Response**: We would like to thank the reviewer for the valuable comments to improve the quality of the manuscript. Mode in SVC-H algorithm, represent that the target class with highest vote will be the final prediction and argmax in SVC-S, represent that the class with highest average probability will be final prediction.

3.3 Fig. 8 (h), (i), (j) - rows of confusion matrices are ordered in different way than in the rest of models. This should be clarified and organized in unified way (the same classes should have the same row number designations in case of all models/matrices. E.g. Fig. 8 (h), (i), (j) row 0 include 35 hits while other confusion matrices in Fig 8 include 35 hits in row number 3. Such changes in class identifiers make this figure hard to understand.
This problem should be removed.

**Response**: We would like to thank the reviewer for the valuable comments. The order is the same for all confusion matrices. The difference which you can see is because of separate data splitting for machine learning and deep learning models. When splitting the dataset we used the shuffle parameter which changes the number of instances for target classes but in both cases, the total numbers of test samples are the same which is 230. We did splitting separately for deep learning models because you can see the last layer of deep learning have 5 neurons so we have to feed target data after converting it into five variables we did splitting again after the conversion of data for deep learning models and shuffled parameters change the count of for each category in the test dataset.

3.4 Sums of the same rows (the same classes) are not equal between matrices in Fig 7.
If this is not an error then it should be explained in more clear way.

**Response**: We would like to thank the reviewer for the valuable comments. We did the separate splitting for both machine learning and deep learning models, because of this there is a difference which we clearly mention in the manuscript and make proposed justification in the 3.2 response.

3.5 Authors write: "All experiments were performed using a Corei7, a 12th generation Dell machine with a Windows operating system."
Are the names of the hardware producer, processor and operating system important for analysis of the results? If yes, this should be clearly discussed within teh text. If not, then not needed information should be removed.

**Response**: We express our gratitude to the reviewer for their valuable feedback. We have included the details of our experimental setup. These details are crucial for the reproducibility of our results as we not only measured the model's performance in terms of accuracy but also in terms of time.

3.6 Authors write: "The rejection is based on the P-value and alpha value, which in this study was set at 0.5 for all scenarios."

Alpha value equal 0.5 represents very weak test. Maybe Authors meant 0.05?
It should be given what was the number of degrees of freedom during calculation of t-statistics? 229?
Why the p-value in case of all comparisons is the same? Is this an effect of imprecision of IEEE754 format used during calculations?

**Response**: We would like to thank the reviewer for the valuable comments. According to reviewer suggestion we did experiment with p value 0.05 and also add the degree of freedom for each comparison. Still with this 0.05 p value our proposed technique reject null hypothesis which show that our proposed approach is statistically significant in comparison with other approaches for disease diagnostics.
