# Supplementary material for: SSC: The novel self-stack ensemble model for thyroid disease prediction
Source: PLoS One. 2024 Jan 3;19(1):e0295501. doi: 10.1371/journal.pone.0295501 (PMC10763970; doi:10.1371/journal.pone.0295501)
Supplement: Attachment — Submitted filename: SSC R4.docx [file pone.0295501.s003.docx]

**RESPONSE TO REVIEWERS**

**Manuscript ID:** PONE-D-22-32955

**Article Title:** “The Novel Self-Stack Ensemble Model for Thyroid Disease Prediction”

**Author:** Ji Sheng-jun

Dear Editor,

First, we are extremely grateful to the editor and all the reviewers who conducted a rigorous and constructive review of our article number PONE-D-22-32955. Certainly, all the review points were very well directed. We have incorporated suggestions and guidance provided by the reviewer and made a careful revision of the manuscript. This has certainly, raised the quality of this research work and will make it a valuable contribution to the community.

Please see our responses (in blue color) in line with the reviewers’ comments in black color.

Best regards,

Ji Sheng-jun

**Reviewer #4:**

**Concern 1:** >>> 2. Presentation problems: 2.1 Table 11, Table 13, Table 14: units are not presented in the header of the table (accuracy in [%] or [1]).
This was indicated previously but not fixed. ACC=0.97 can mean e.g. 0.97% or 97%
**Response**: We would like to thank the reviewer for raising concerns. Here, we added clear details about score units, as these accuracy, precision, recall, and F1 scores are standard parameters, so their values are always between 0 and 1. Here, 0 is the lowest score and 1 is the highest score. There will be no unit with them.

We added this explanation in the manuscript for readers in the evaluation matrix section.

**Concern 2:** 2.2 Fig. 9, Fig. 5, Fig 6, Fig 2: Results should be presented with error bars.

**Response**: We would like to thank the reviewer for the valuable comments. We already responded to these comments in our previous response. Again we clarify this comment point by point. We also added one more figure (Figure 10) to illustrate error bars.

- Fig 2: This figure is already a bar chart but we can’t convert it into an error bar. Because of its feature importance score always one value corresponds to one feature.
- Fig 5 and Fig 6: We convert these figures into bar charts as shown in Figure 9. These two figures consist of accuracy and error graphs so we convert them into bar charts and illustrate them in one graph.
- Fig 9: We added the error rate in Fig 9 and error bars in Fig 10 and updated the manuscript.

**Concern 3:** Fig. 2, Fig. 5, Fig. 6: Not fixed.

**Response**: We fixed these figures and converted them according to the reviewer concern.

**Concern 4:** Fig. 9 - modified but not fixed: Classification Error (1 - Classification Accuracy) was added/presented by the Authors within Fig. 9 but this is not what was meant by the Reviewer. Measurement errors need to be presented instead, e.g. for p-value=0.95.
If Authors want to use bar chart then bar chart with error bars should be presented.

Please see the exmple discussions of the measurement error/measurement uncertainty and related confidence intervals:
https://blogs.sas.com/content/iml/2019/10/09/statistic-error-bars-mean.html
<https://www.r-bloggers.com/2021/06/error-bar-plot-in-r-adding-error-bars-quick-guide/>

**Response**: Figure 10, illustrates SD values for different classifiers and provides insights into the variability of accuracy scores when applied to both original and down-sampled data. It also shows the error rate lower and upper bounds. These values indicate the stability of classifier performance across multiple runs or datasets, with lower values suggesting more consistent results.

**Concern 5:** 2.3 Table 15: There is no point presenting columns with the same value in all rows of the table. Such information can be given in the title of the table or below the table.
Not fixed. After modifications Table 15 still includes three columns (df, P-Value, Null Hypothesis) which include the same values in each row.

**Response**: We remove the values and mention the values in the text.

**Concern 6:**2.4 Algorithm 2:
"D = Number of base classifier." => "D = Number of base classifiers."
Are Authors sure that plural version would not be more accurate?

**Response**: Yes, the number of base classifiers is correct and we update it accordingly.

**Concern 7:** 2.5 Fig. 5, Fig. 6: what is the meaning of "Val_Accuracy", "Val_Precision", "Val_Recall", "Val_F1", "Val_Loss", "Val_MSE" within legends of included charts?
What is the difference e.g. between "Accuracy' and ""Val_Accuracy"? This should be explained within the title of the figure.
Authors write in their response:"‘Val_Accuracy’ represent the accuracy on the validation set, it is also similar to other
evaluation matrixes. We added the description of it in the updated manuscript."

The information was added within the text. It would be better to add relevant information within titles of Fig 5 and Fig 6.
The Reader can have a problem to find this information while analysing those figures.

**Response**: We updated the manuscript according to the reviewer's suggestion and added details in the text and captions.

The terms 'Accuracy' and 'Val_Accuracy' in Figures 5 and 6 correspond to the accuracy metrics computed on the training and validation datasets, respectively. This same concept extends to other evaluation metrics, where 'val_precision' represents validation precision, 'val_recall' stands for validation recall, and 'val_F1_Score' stands for the validation F1 score.

**Concern 8:** 3.1 Why the time in Table 13 is given with varying precision for different models?
What was the precision of time measurement setup? SD or confdence intervals should be given with measured value.

**Response**: Reviewers' concern is valid, and we would like to address it. The variation in processing time can be attributed to differences in model architectures. For instance, linear models like Support Vector Classifier (SVC) demand more computational resources and time to find an optimal hyperplane, whereas models like Random Forest (RF), Logistic Regression (LR), and Gradient Boosting Machine (GBM) have less complex architectures compared to SVC. Therefore, the time precision is entirely dependent on the specific model's architecture.

**Concern 9:** 3.2 Fig. 8 (h), (i), (j) - rows of confusion matrices are ordered in different way than in the rest of models. This should be clarified and organized in unified way (the same classes should have the same row number designations in case of all models/matrices. E.g. Fig. 8 (h), (i), (j) row 0 include 35 hits while other confusion matrices in Fig 8 include 35 hits in row number 3. Such changes in class identifiers make this figure hard to understand.
This problem should be removed.
Authors gave the explanation within their response to the Reviewer.
The explanation should be added within the text of the manuscript it must be available to the Reader.

**Response**: We added these lines in the manuscript according to the reviewer suggestion.

Figures 7 and 8 show the confusion matrix for both machine learning and deep learning models on the same size of test data. We have done separate data splitting (Training and Testing sets) for machine learning and deep learning models. When splitting the dataset, we used the shuffle parameter which changes the number of instances for each target class for both machine and deep learning models but in both cases, the total number of test samples is the same which is 230. We did splitting separately for deep learning models because you can see the last layer of deep learning has 5 neurons so we have to feed target data after converting it into five variables we did splitting again after the conversion of data for deep learning models and shuffle parameters change the count of for each category in the test dataset.

**Concern 10:** 3.3 Sums of the same rows (the same classes) are not equal between matrices in Fig 7. If this is not an error then it should be explained in more clear way.
Authors gave the explanation within their response to the Reviewer.
The explanation should be added within the text of the manuscript it must be available to the Reader.

**Response**: This is the same concern as above and we explain it in the text. This difference in the number of instances in each row is because of reshuffle parameters in the train test split. This shuffle makes validation more strong when you have an imbalance or a small dataset.

We added an explanation for this in the manuscript as above for readers.
