# Supplementary material for: SSC: The novel self-stack ensemble model for thyroid disease prediction
Source: PLoS One. 2024 Jan 3;19(1):e0295501. doi: 10.1371/journal.pone.0295501 (PMC10763970; doi:10.1371/journal.pone.0295501)
Supplement: Attachment — Submitted filename: SSC5.docx [file pone.0295501.s004.docx]

**RESPONSE TO REVIEWERS**

**Manuscript ID:** PONE-D-22-32955

**Article Title:** “The Novel Self-Stack Ensemble Model for Thyroid Disease Prediction”

**Author:** Ji Sheng-jun

Dear Editor,

First, we are extremely grateful to the editor and all the reviewers who conducted a rigorous and constructive review of our article number PONE-D-22-32955. Certainly, all the review points were very well-directed. We have incorporated suggestions and guidance provided by the reviewer and made a careful revision of the manuscript. This has certainly, raised the quality of this research work and will make it a valuable contribution to the community.

Please see our responses (in blue color) in line with the reviewers’ comments in black color.

Best regards,

Ji Sheng-jun

Editor Comments:

Thank you for submitting your manuscript to PLOS ONE. It was analyzed by me as an Academic Editor (Reviewer #4). After careful consideration, we feel that it has merit but does not fully meet PLOS ONE’s publication criteria as it currently stands. Therefore, we invite you to submit a revised version of the manuscript that addresses the points raised during the review process.

In particular:

- reporting of statistical results should be improved,

We have updated the manuscript by incorporating the reviewer's suggested details on the power of the test.

- selection of statistical tests should be backed up by the presented data,

The data used for the test is already in the manuscript, as we utilized the model evaluation scores in the statistical analysis. So every model evaluation is present in the results section.

- indicated presentation and language problems should be removed.

We thoroughly reviewed the manuscript and corrected issues according to the reviewer's suggestions.

Reviewer #4:

>>> 1. Language problems:
1.1 Authors write: "We did splitting separately for deep learning models because you can see the last
layer of deep learning has 5 neurons so we have to feed target data after converting it
into five variables we did splitting again after the conversion of data for deep learning
models and shuffle parameters change the count of for each category in the test dataset."

Authors should not mix plain language with scientific language ("you can see").
Additionally - this sentence should be split into shorter and more clear ones. The current form is hard to understand.
Response: We would like to thank the reviewer for their valuable comments and suggestions. The manuscript has been updated in accordance with these suggestions, and the changes can be found in the updated version
>>> 2. Presentation problems:
Fig 10. (a) vertical axis title is improper (names of classifiers are under horizontal axis)
Fig 10. (b) vertical axis title is not as title of the figure suggests.

Response: The reviewer raised valuable points in the manuscript, and we have updated the manuscript to address their concerns. Updated figures can be found in the revised manuscript.


>>> 3. Other problems:
3.1 Reporting of statistical analysis is not complete:
- what is the sample size? (How many times each measurement was repeated? How many times 10-fold CV was repeated?)
- Authors report statistical importance, but what is the size of the observed effect (e.g. Cohens delta?) ?
- What is the estimated power of the performed test? (knowing the Cohens delta and number of samples you can use e.g. "Statistical Nomograph for Sample Size Estimation by Richard N. MacLennan"). Power of the test should be not lower than 0.7. This will allow to check if the number of measurments (sample size) is big enough.

**Response**: We would like to express our gratitude to the reviewer for their valuable comments. To determine the statistical power of our test, we utilized the suggested methods and an online power calculator [1]. Our sample size is 16, Cohen's effect size is 0.8, and the significance level is set at 0.05. The sample population consists of a normal distribution. The test results provide a statistical power of 0.169, with critical values of -1.96 and 1.96 based on the standard normal distribution, where the standard distribution shift is 2.263.

1- <https://www.statskingdom.com/32test_power_t_z.html>

3.2 Selection of the statistical test is not backed up by the presented data and Authors do not present information if the analysed data meet assumptions needed to use this test (normal distribution, homogeneity of variance). What statistical tests of normality and variance homogeneity were used to validate this?
**Response**: We used a normal distribution for the test, and all other parameters in the test were included in the updated manuscript. However, the reviewer raised concerns regarding the quality of the data, as we utilized model results for performing a statistical T-test. This means that the results from the models, including accuracy, per-class (precision, recall, F1 score), and their average, were subjected to a T-test to demonstrate their significance. We compared the results of the SSC model with those of all other models, as displayed in Table 15.

3.3 Construction of the statistical test seems to be not valid. Authors are using solution which is typically used as a post-hoc test. Maybe Authors could use Friedmans ANOVA as a main test?
Please see e.g. [A] and [B] for details.
[A] Janez Demsar, Statistical Comparisons of Classifiers over Multiple Data Sets, Journal of Machine Learning Research 7, 2006
[B] Dietterich T.G., Approximate statistical tests for comparing supervised classification learning algorithms, Neural Comput 10(7), 1998

**Response**: We would like to express our gratitude to the reviewer for their valuable comments. Indeed, the reviewer's point is accurate, as we deployed a T-test on the model results to assess the statistical differences in model performance. The study [B] recommended applying these tests to model predictions, which can help obtain a larger sample for statistical analysis. We conducted statistical tests on the evaluation scores to emphasize the significance of the models over others based on their performance scores. In this study, our main contribution is classification and proposing a model. Our aim is to highlight the significance of the proposed models and we adopt a very simple approach to apply statistical Tests rather than adopting complex approach.
